# A Mixed-Methods Study on Topical Fluoride Beliefs and Refusal Behaviors for Caregivers of Children with Special Health Care Needs

**DOI:** 10.1007/s10995-023-03806-1

**Published:** 2023-11-15

**Authors:** Madelyn Koh, Darragh Kerr, Courtney M. Hill, Donald L. Chi

**Affiliations:** Department of Oral Health Sciences, School of Dentistry, University of Washington, B530D, Box 357475, 1959 NE Pacific St., Seattle, WA 98195 USA

**Keywords:** Fluoride, Topical fluoride, Health belief model, Dental care for the disabled, Children with disabilities, Mixed methods, Treatment refusal

## Abstract

**Objective:**

To understand topical fluoride-related beliefs and refusal behaviors for caregivers of children with special health care needs (CSHCN).

**Methods:**

This was an explanatory sequential mixed methods study. For the quantitative analyses, we surveyed 520 caregivers to (a) compare fluoride-related beliefs between caregivers of CSHCN and caregivers of healthy children and (b) evaluate the association between special health care need (SHCN) status and topical fluoride refusal. We used logistic regression models to generate unadjusted odds ratios, confounder-adjusted odds ratios (AOR), and 95% confidence intervals (CI). For the qualitative analyses, we interviewed 56 caregivers who refused or were hesitant about topical fluoride. Data were coded deductively and compared by SHCN status to an existing conceptual model of topical fluoride refusal.

**Results:**

In the quantitative analysis, 41.3% of caregivers refused or thought about refusing topical fluoride. There were no significant differences in fluoride beliefs by SHCN status (*p*-values > 0.05) nor was there a significant association between SHCN status and topical fluoride refusal (AOR: 0.65, 95% CI 0.37–1.14; *p* = 0.13). In the qualitative analysis, the relative importance of each domain of the conceptual model was similar between the caregiver groups. Two differences were that all caregivers of CSHCN thought fluoride was unnecessary and wanted to keep chemicals out of their child’s body.

**Conclusions for Practice:**

While caregivers of CSHCN were not more likely to refuse topical fluoride than caregivers of healthy children, there may be important differences in the underlying reasons for refusing topical fluoride.

**Supplementary Information:**

The online version contains supplementary material available at 10.1007/s10995-023-03806-1.

## Significance

*What is already known on this subject?* CSHCN are at increased risk for tooth decay. Topical fluoride helps prevent tooth decay, but large numbers of caregivers refuse topical fluoride. A conceptual model of caregiver topical fluoride refusal has been developed, but its applicability to caregivers of CSHCN has not yet been evaluated.

*What this study adds?* This study provides empirical insight into fluoride refusal behaviors for caregivers of CSHCN. Caregivers of CSHCN may have different reasons for refusing topical fluoride. Findings from this study will help guide the development of tailored communication approaches for caregivers of CSHCN who refuse or may be hesitant about topical fluoride.

**Supplementary Information:**

The online version contains supplementary material available at 10.1007/s10995-023-03806-1.

## Introduction

One-in-four US families has a child with special health care needs (SHCN) (HRSA, 2020). Dental care is the most prevalent unmet need for children with SHCN (CSHCN), and dental needs are greater for CSHCN from low-income families and those with more complex needs (Lewis, 2009). Furthermore, many CSHCN are prescribed sugary medications, given sweets to manage behaviors, have disabilities that can make toothbrushing difficult, and encounter barriers to dental care (Campanaro et al., 2014; Liu et al., 2010). As a result, subgroups of CSHCN are at increased risk for tooth decay (Chi et al., 2013).

While topical fluoride prevents tooth decay (Weintraub et al., 2006), caregiver refusal of topical fluoride has become a growing problem (Chi, 2017; Chi & Basson, 2018). Topical fluoride behaviors are thought to be on a continuum, with acceptance at one end, refusal at the other, and varying degrees of hesitancy along the continuum (Chi, 2017). One preliminary study reported that 12.7% of caregivers refused topical fluoride for their child during dental visits (Chi, 2014). The proportion of CSHCN caregivers who refuse or are hesitant about topical fluoride is unknown.

The reasons for topical fluoride hesitancy and refusal are not fully understood. One potential explanation is incomplete knowledge about fluoride (Chi et al., 2018). This could lead not only to refusal of topical fluoride during dental visits but also to avoidance of other common fluoride sources including water and toothpaste (Ko & Chi, 2023). Caregivers of CSHCN are known to actively seek information prior to making healthcare decisions (Du et al., 2019), which could expose caregivers to Internet-based misinformation- and disinformation (Hoffman et al., 2019). Between 2009 and 2017, 60% of water fluoridation mentions on Twitter were negative compared to 15% that were positive (Oh et al., 2020). Some caregivers believe fluoride is a neurotoxin that leads to lower IQ, autism, cancer, and other diseases (Choi et al., 2012; Strunecka & Strunecky, 2019). More recently, researchers have sought to identify the reasons for topical fluoride hesitancy and refusal. Based on qualitative interviews with 56 caregivers, Chi and colleagues developed a conceptual model with six domains to explain why caregivers are hesitant about topical fluoride (Chi et al., 2023).

Vaccine refusal is a documented correlate of topical fluoride refusal (Chi, 2014). The factors driving vaccine refusal may be similar to those that lead to topical fluoride refusal. Caregivers of CSHCN are more likely to refuse childhood vaccines than caregivers of healthy children (Cody & Lerand, 2013; Greenwood et al., 2013). One of the underlying concerns is that vaccines are unsafe and lead to conditions like autism (Abu Kuwaik et al., 2014; Dannetun et al., 2005; Roberts et al., 2015), a belief that has its origins in a now retracted publication from the 1990s (Eggertson, 2010).

Studies on topical fluoride refusal focusing on caregivers of CSHCN are limited. One study from Italy found that CSHCN have lower levels of fluoride exposure (Bagattoni et al., 2021). Another study from Singapore found that caregivers of CSHCN and caregivers of healthy children reported similar rates of refusal of silver diamine fluoride, which is another type of topical fluoride treatment (Hu et al., 2020). One US study reported that while caregivers of CSHCN had similar attitudes toward fluoridated products as caregivers of neurotypical children, the former were significantly less likely to use fluoridated toothpaste for their child (Capozza & Bimstein, 2012). Current literature in the US suggests that CSHCN caregivers are more likely to refuse topical fluoride because of safety concerns (Rada, 2010), but there is no empirical evidence for this. Thus, it is not clear whether caregivers of CSHCN have different beliefs regarding topical fluoride, whether they are more likely to refuse topical fluoride than caregivers of healthy children, and if the reasons for refusal or hesitancy are different.

There were three goals in this mixed methods study. The first goal was to compare fluoride-related beliefs for caregivers of CSHCN and caregivers of healthy children. The second goal was to evaluate the association between SHCN status and topical fluoride refusal. The third was to determine whether the reasons for topical fluoride refusal were different by SHCN status.

## Methods

### Study Design

This was an explanatory sequential mixed methods study involving secondary data collected by survey and interview from caregivers of CSHCN and healthy children (Fetters et al., 2013). The study was conducted in accordance with prevailing ethical principles and approved by the University of Washington Institutional Review Board.

### Part 1: Quantitative Study

#### Participants

We recruited caregivers at five dental clinics in children’s hospitals or university-based pediatric dental clinics in the US. Survey participants were also recruited through social media, flyers, private dental practices, and naturopathic medicine practices. All participants were required to be at least 18 years old, provide informed consent, able to read and understand English, and to be a caregiver of a child under the age of 18 years. A sample size of at least 500 caregiver survey respondents was based on power calculations for the primary intent of the survey data, which was to conduct psychometric testing of a fluoride hesitancy identification tool (Carle et al., 2022; Edwards et al., 2023). We did not track survey participation rates.

#### Study Procedures

We piloted, revised, and finalized an 85-item questionnaire that included questions on caregiver beliefs about topical fluoride, history of fluoride hesitancy or refusal, and demographics (Online Appendix). We sent potential participants a postcard or an email link to an online REDCap survey. Additional participants completed the online survey on study tablets at one of the study sites or accessed the survey link through QR codes placed on social media posts or study flyers. The survey was administered from November 2020 to April 2021. For caregivers with multiple children, the youngest child was designated as a referent. Before the survey, participants were asked to read and accept a written consent statement. Written documentation of consent was not obtained. After completing the survey, caregivers had the option to enter a raffle to win an Apple iPad or a pair of electric toothbrushes.

#### Independent Variable

The independent variable was being a caregiver of a child with SHCN (no/yes). Caregivers were classified as having a child with SHCN if they reported their child required a medication other than vitamins prescribed by a doctor (Carle et al., 2011).

##### Primary Outcome Variables

The primary outcome variables were topical fluoride beliefs, which were measured with six items (Table 1). Response options were on a 0–10 scale or a four-category Likert-like response. For the statistical analyses, all responses were dichotomized.

**Table 1:** Survey items measuring caregiver beliefs about topical fluoride

Survey item	Response options	Operationalization for analyses
On a scale of 0 to 10, how concerned are you about topical fluoride for your child?	0–10	0 = not concerned 1–10 = some degree of concern
I think topical fluoride is harmless for my child	1 = strongly agree 2 = agree 3 = disagree 4 = strongly disagree	1–2 = agree 3–4 = disagree
I think there is enough proof that topical fluoride is safe for my child		
I am concerned topical fluoride may cause learning problems for my child	1 = extremely concerned 2 = somewhat concerned 3 = slightly concerned 4 = not at all concerned	1–3 = concerned 4 = not concerned
I am concerned topical fluoride may cause my child to have autism		
I am concerned topical fluoride may hurt my child’s IQ		

##### Secondary Outcome Variable

The secondary outcome, topical fluoride refusal, was measured with the following item: “Regarding topical fluoride in general for your child, which statement below best describes you.” The five-category response options included: (1) “I always say no”, (2) “Most of the time I say no”, (3) “Sometimes I say no”, (4) “I say yes, but I have thought about saying no”, or (5) “I always say yes.” Caregivers who responded 1–4 were classified as expressing some degree of topical fluoride hesitancy or refusal and those who responded 5 were classified as accepting topical fluoride.

##### Confounding Variables

There were six confounders: caregiver age, gender, race, ethnicity, education, and household income. Age was modeled as a continuous variable. There were three gender categories (male, female, nonbinary/other). Caregiver race was a self-reported nine-category variable corresponding to the US Census Bureau categories (white, Black, Asian, American Indian or Alaskan Native, Native Hawaiian or other Pacific Islander, other, multiple race). Ethnicity was defined as Hispanic, Latino, or Spanish origin (no/yes). There were five education categories, ranging from “less than a high school diploma” to “more than a four-year college degree.” Income was a seven-category variable.

#### Statistical Analysis

Descriptive statistics were generated for the study population and either the independent *t*-test (continuous variables) or chi-square test (categorical variables) was used to compare the distribution of demographic characteristics by SHCN status. We generated the distribution of the primary and secondary outcome variables for the overall study population and by SHCN status. Logistic regression models were used to generate unadjusted odds ratios (OR) and confounder-adjusted odds ratios (AOR) with corresponding 95% confidence intervals (CI) for the associations between SHCN status and (a) topical fluoride beliefs and (b) topical fluoride refusal (*α* = 0.05). Participants with missing data were excluded from the regression models. All analyses were completed using SPSS v 27.

### Part 2: Qualitative Study

#### Participants

Caregivers were eligible if they were at least 18 years old, able to communicate in English, the caregiver of a child under the age of 18 years, and if they did not decline topical fluoride exclusively for financial reasons. Caregivers who answered ≥ 2 to the screening question “On a scale of 1 to 10, with 1 being not opposed at all and 10 being totally opposed, how opposed are you to topical fluoride for your child or any of your children?” were eligible for the study. We used billing codes and health records from two pediatric dentistry clinics in Washington State to identify children who did not receive topical fluoride during a routine dental visit between August 2016 and September 2018. Additional caregivers were identified through private practice clinic referrals, social media, personal networks of study team members, and through snowball sampling. Trained research assistants contacted 513 caregivers of these children by telephone to verify eligibility. We were able to reach 173 caregivers, of whom 56 were not interested and 12 did not speak English. Of the remaining 95 caregivers, 23 were not eligible. Of the remaining 76 caregivers, 56 completed an interview.

#### Data Collection, Management, and Analysis

**Fig. 1:**
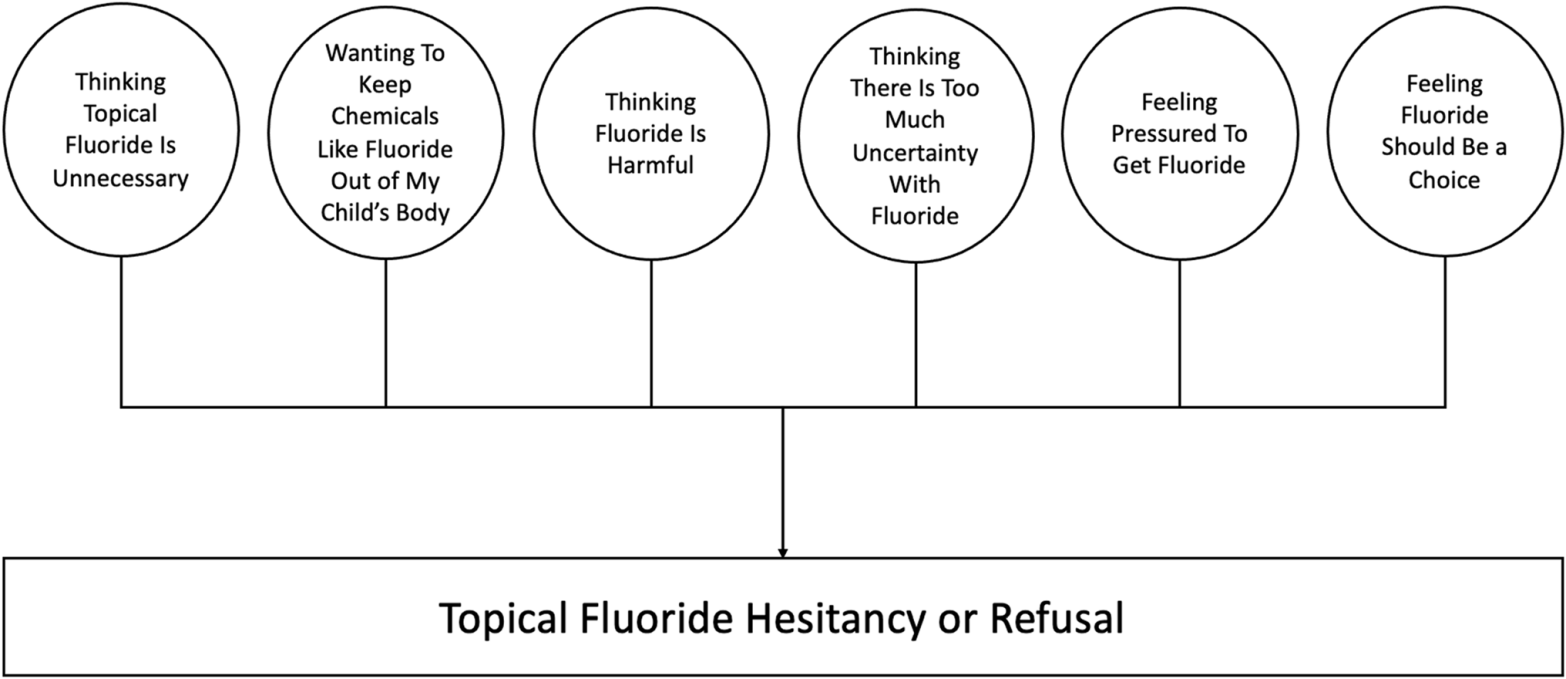
Conceptual six-domain model of topical fluoride hesitancy or refusal

Interview data were collected using procedures described previously with informed consent obtained prior to the start of the interview (Chi et al., 2023). Caregiver participants were recruited and interviewed until saturation on themes was reached. The interview transcripts were manually reviewed and collated into two groups based on whether the caregiver voluntarily stated during the interview that their child has a SHCN. We generated descriptive statistics on the interview population and compared characteristics by SHCN status (yes/no) using the t-test or chi-square test (α = 0.05). Based on previous qualitative work (Chi et al., 2023), we created a codebook with 21 categories organized into six domains, with each domain indicating a reason for refusing topical fluoride: (1) thinking topical fluoride is unnecessary; (2) wanting to keep chemicals like fluoride out of my child’s body; (3) thinking fluoride is harmful; (4) thinking there is too much uncertainty with fluoride; (5) feeling pressured to get fluoride; and (6) feeling fluoride should be a choice (Fig. 1). Transcripts were coded using a deductive coding approach and content analytic techniques (Corbin & Strauss, 2008; Krippendorf, 1980). A unit of data consisted of one complete thought. Units were grouped together based on common themes in the category. To ensure accuracy, we used comparative analysis to compare the unitized data across the categories (Corbin & Strauss, 2008). Coding discrepancies were addressed and resolved through debriefing between coders. After coding, caregiver responses within each category and domain were calculated as percentages. While caregivers could be represented across multiple categories, responses from each caregiver were counted only once per category. All quotes reported were from caregivers of CSHCN. Analyses were completed using SPSS v 27.

## Results

### Part 1: Quantitative Study

#### Descriptive Statistics

Among the 520 surveyed caregivers, 87 (16.7%) reported having a child with SHCN and 433 (82.3%) had a healthy child (Table 2). Caregivers of CSHCN were significantly older than caregivers of healthy children (44.6 years and 41.1 years, respectively; *p* < 0.001). There were no other significant differences between caregiver groups.

**Table 2:** Caregiver or household sociodemographic characteristics corresponding to surveyed caregivers of children with SHCN and caregivers of healthy children (*N* = 520)

Caregiver or household characteristic	All caregivers	Caregivers of children with SHCN	Caregivers of healthy children	*p*-value^a^
	(*N* = 520)	(*N* = 87)	(*N* = 433)	
	*n* (%) or mean (SD, range)	*n* (%) or mean (SD, range)	*n* (%) or mean (SD, range)	
Age	41.7 (SD 8.0, 18–72)	44.6 (SD 9.1, 18–72)	41.1 (SD 7.7, 21–71)	< 0.001***
Number of children under 18 years^b^	2.07 (SD 1.0, 0–7)	1.7 (SD 0.8, 1–5)	2.2 (SD 1.0, 0–7)	< 0.001***
Age of youngest child^c^	6.97 (SD 4.4, 0–18)	9.2 (SD 4.6, 0–17)	6.5 (SD 4.2, 0–18)	0.002**
Gender				0.61
Male	82 (15.8%)	13 (14.9%)	69 (15.0%)	
Female	405 (77.9%)	70 (80.5%)	335 (77.4%)	
Nonbinary/other	3 (0.6%)	1 (1.1%)	2 (0.5%)	
Missing	30 (5.8%)	3 (3.4%)	27 (6.2%)	
Race				0.25
White	303 (58.3%)	62 (71.3%)	241 (55.7%)	
Black	29 (5.6%)	5 (5.7%)	24 (5.5%)	
Asian	86 (16.5%)	8 (9.2%)	78 (18.0%)	
American Indian or Alaskan Native	6 (1.2%)	1 (1.1%)	5 (1.2%)	
Native Hawaiian or other Pacific Islander	4 (0.8%)	2 (2.3%)	2 (0.5%)	
Other	33 (6.3%)	4 (4.6%)	29 (6.7%)	
Multiple races	24 (4.6%)	2 (2.3%)	22 (5.1%)	
Missing	35 (6.7%)	3 (3.4%)	32 (7.4%)	
Ethnicity				0.34
Hispanic, Latino, or Spanish origin	50 (9.6%)	6 (6.9%)	44 (10.2%)	
Not Hispanic, Latino, or Spanish origin	435 (83.3%)	75 (86.2%)	358 (82.7%)	
Missing	37 (7.1%)	6 (6.9%)	31 (7.2%)	
Education				0.88
Less than high school diploma	14 (2.7%)	2 (2.3%)	12 (2.8%)	
High School or GED^d^ credential equivalent	45 (8.6%)	7 (8.0%)	38 (8.8%)	
Some college or 2-year degree	119 (22.8%)	20 (23%)	99 (22.9%)	
4-year college degree	130 (24.9%)	26 (29.9%)	103 (23.8%)	
More than 4-year college degree	183 (35.0%)	29 (33.3%)	153 (35.3%)	
Missing	32 (6.1%)	3 (3.4%)	28 (6.5%)	
Household income				0.61
Less than $15,000	28 (5.4%)	4 (4.6%)	24 (5.5%)	
$15,000–$25,000	37 (7.1%)	5 (5.7%)	32 (7.4%)	
$25,000–$50,000	93 (17..9%)	13 (14.9%)	80 (18.5%)	
$50,000–$75,000	75 (14.4%)	17 (19.5%)	58 (13.4%)	
$75,000–$100,000	60 (11.5%)	9 (10.3%)	51 (11.8%)	
$100,000–$150,000	93 (17.9%)	20 (23.0%)	73 (16.9%)	
$150,000 or more	87 (16.7%)	13 (14.9%)	74 (17.1%)	
Missing	47 (9.0%)	6 (6.9%)	41 (9.5%)	

*p < .05 **p < .01 *** *p* < .001

^a^Significance testing was conducted with the t-test for continuous variables (e.g., caregiver age) and chi-square test for categorical variables

^b^Number of children under the age of 18 years that live in the same household

^c^Age of youngest child under the age of 18 years that live in the same household

^d^GED, General Educational Development

#### Beliefs about Topical Fluoride

Caregiver beliefs about topical fluoride are summarized in Table 3. In the confounder-adjusted models, there were no significant differences in topical fluoride beliefs by SHCN status.

**Table 3:** Unadjusted and adjusted regression analyses of caregiver beliefs about topical fluoride for caregivers of CSHCN and caregivers of healthy children

Topical fluoride beliefs	Caregivers of all children *n* (%)	Caregivers of CSHCN^b^ *N* = 87 *n* (%)	Caregivers of healthy children *N* = 433 *n* (%)	Unadjusted odds ratio^d^ (95% CI)^c^	*p*-value	Adjusted^a^ odds ratio^d^ (95% CI)^c^	*p*-value
On a scale of 0–10, how concerned are you about topical fluoride for your child?							
Some degree of concern	259 (49.8%)	44 (50.6%)	215 (49.7%)	1.02 (0.65–1.62)	0.92	1.02 (0.61–1.73)	0.93
Not concerned	258 (49.6%)	43 (49.4%)	215 (49.7%)	–	–	–	–
Missing	3 (0.6%)	0	3 (0.7%)				
I think topical fluoride is harmless for my child							
Agree	325 (62.5%)	61 (70.1%)	264 (61.0%)	1.50 (0.90–2.48)	0.12	1.78 (0.99–3.19)	0.05
Disagree	187 (36.0%)	25 (28.7%)	162 (37.4%)	–	–	–	–
Missing	8 (1.5%)	1 (1.1%)	7 (1.6%)				
I think there is enough proof that topical fluoride is safe for my child							
Agree	386 (74.2%)	72 (82.8%)	314 (72.5%)	1.70 (0.93–3.08)	0.08	1.86 (0.94–3.67)	0.07
Disagree	126 (24.2%)	15 (17.2%)	111 (25.6)	–	–	–	–
Missing	8 (1.5%)	0	8 (1.8%)				
I am concerned topical fluoride may cause learning problems for my child							
Concerned	168 (32.3%)	25 (28.7%)	143 (33.0%)	0.80 (0.48–1.33)	0.39	0.96 (0.53–1.72)	0.88
Not concerned	340 (65.4%)	61 (70.1%)	279 (64.4%)	–	–	–	–
Missing	12 (2.3%)	1 (1.1%)	11 (2.5%)				
I am concerned topical fluoride may cause my child to have autism							
Concerned	90 (17.3%)	13 (14.9%)	77 (18.2%)	0.80 (0.42–1.51)	0.49	0.95 (0.43–2.07)	0.89
Not concerned	418 (80.4%)	73 (83.9%)	345 (81.8%)	–	–	–	–
Missing	12 (2.3%)	1 (1.1%)	11 (2.5%)				
I am concerned topical fluoride may hurt my child’s IQ							
Concerned	127 (24.4%)	17 (19.5%)	110 (26.1%)	0.71 (0.40–1.26)	0.24	0.73 (0.37–1.44)	0.36
Not concerned	380 (73.1%)	68 (78.2%)	312 (73.9%)	–	–	–	–
Missing	13 (2.5%)	2 (2.3%)	11 (2.5%)				

^a^Model was adjusted for the following confounders: caregiver age, race, ethnicity, gender, education, and household income

^b^CSHCN, Children with Special Health Care Needs

^c^95% confidence interval

^d^Odds ratio is for caregivers of children with special health care needs (reference group: caregivers of healthy children)

#### Topical Fluoride Refusal

The prevalence of topical fluoride refusal was 34.5% for caregivers of CSHCN and 43.3% for caregivers of healthy children. There was no significant difference in topical fluoride refusal by SHCN status in the unadjusted and confounder-adjusted models (unadjusted OR: 0.70; 95% CI 0.43–1.14; *p* = 0.15; AOR: 0.65; 95% CI 0.37–1.14; *p* = 0.13, respectively). Among caregivers of CSHCN, 7.0% always said no to topical fluoride, 3.5% said no most of the time, 9.3% sometimes said no, 15.1% said yes but thought about saying no, and 65.1% always said yes, which was not statistically different from caregivers of healthy children (*p* = 0.73).

### Part 2: Qualitative Study

#### Descriptive Statistics

Of the 56 interviewed caregivers, 12 reported having a child with SHCN (Table 4). Caregivers of CSHCN were significantly older than caregivers of healthy children (47.4 years and 40.5 years, respectively; *p* = 0.03). There were no other differences by SHCN status.

**Table 4:** Caregiver or household sociodemographic characteristics of interviewed caregivers of CSHCN and caregivers of healthy children (*N* = 56)

Caregiver or household characteristic	All caregivers	Caregivers of CSHCN	Caregivers of healthy children	*p*-value^a^
	(*N* = 56)	(*N* = 12)	(*N* = 44)	
		*n* (%) or mean (SD, range)	*n* (%) or mean (SD, range)	
Age	42.0 (SD 9.98, 29–79)	47.4 (SD 14.6, 34–79)	40.5 (SD 7.9, 29–63)	0.03*
Number of children under 18 years^b^	2.0 (SD 1.3, 1–9)	1.9 (SD 1.4, 1–5)	2.0 (SD 1.3, 1–9)	0.15
Age of youngest child^c^	7.2 (SD 4.5, 0.8–18)	6.8 (SD 4.3, 1–15)	7.3 (SD 4.6, 0.8–18)	0.37
Gender				0.22
Male	5 (8.9%)	0	5 (11.4%)	
Female	51 (91.1%)	12 (100%)	39 (88.6%)	
Race				0.12
White	32 (57.1%)	11 (91.7%)	21 (47.7%)	
Black	4 (7.1%)	0	4 (9.1%)	
Asian	6 (10.7%)	0	6 (13.6%)	
American Indian or Alaskan Native	2 (3.6%)	0	2 (4.5%)	
Native Hawaiian or other Pacific Islander	0	0	0	
Multiple races	9 (16.1%)	0	9 (20.5%)	
Other	2 (3.6%)	1 (8.3%)	1 (2.3%	
Missing	1 (1.8%)	0	1 (2.3%)	
Ethnicity				0.83
Not Hispanic, Latino or Spanish origin	49 (87.5%)	11 (91.7%)	38 (86.4%)	
Hispanic, Latino or Spanish origin	6 (10.7%)	1 (8.3%)	5 (11.4%)	
Missing	1 (1.8%)	0	1 (2.3%)	
Education				0.86
High School or GED^d^ credential equivalent	2 (3.6%)	0	2 (4.5%)	
Some college or 2-year degree	18 (32.1%)	4 (33.3%)	14 (31.8%)	
4-year college degree	20 (35.7%)	5 (41.7%)	15 (34.1%)	
More than 4-year college degree	16 (28.6%)	3 (25.0%)	13 (29.5%)	
Missing	0	0	0	
Income				0.66
Less than $15,000	5 (8.9%)	2 (16.7%)	3 (6.8%)	
$15,000–$25,000	2 (3.6%)	0	2 (4.5%)	
$25,000–$50,000	7 (12.5%)	2 (16.7%)	5 (11.4%)	
$50,000–$75,000	13 (23.2%)	3 (25.0%)	10 (22.7%)	
$75,000–$100,000	4 (7.1%)	0	4 (9.1%)	
$100,000–$150,000	13 (23.2%)	4 (33.3%)	9 (20.5%)	
$150,000 or more	4 (7.1%)	0	4 (9.1%)	
Missing	8 (14.3%)	1 (8.3%)	7 (15.9%)	

*p < .05 **p < .01 *** *p* < .001

*m* mean, *SD* standard deviation, *CSHCN* children with special health care needs

^a^Significance testing was conducted with a *t*- test for continuous variables (i.e., caregiver age) and a chi-square test for categorical variables (i.e., all other variables)

^b^Number of children under the age of 18 years that live in the same household

^c^Age of youngest child under the age of 18 years that live in the same household

^d^GED, General Educational Development

#### Reasons for Refusing Topical Fluoride

Responses from caregivers of CSHCN were represented across all six domains from the conceptual model (Table 5). Caregivers of CSHCN provided similar reasons for topical fluoride refusal as caregivers of healthy children, but there were observed differences. Below, we provide a summary of findings by domain and report frequencies of responses, highlighting differences by SHCN status.

**Table 5:** Domains and categories of reasons for topical fluoride hesitancy or refusal between caregivers of children with SHCN and caregivers of healthy children

Domains and categories	Caregivers of children with SHCN	Caregivers of healthy children
	(*N* = 12)	(*N* = 44)
Domain 1: Thinking Topical Fluoride Is Unnecessary	12 (100%)	39 (89%)
Thinking my child’s teeth are fine without it	3 (25%)	13 (30%)
Thinking it is not effective	9 (75%)	26 (59%)
Keeping your teeth clean is enough	5 (42%)	26 (59%)
Having a healthy diet is more important	3 (25%)	22 (50%)
Getting fluoride from other sources is enough	4 (33%)	10 (23%)
Domain 2: Wanting To Keep Chemicals Like Fluoride Out Of My Child’s Body	12 (100%)	36 (82%)
Being careful about what goes into my child’s body	4 (33%)	18 (41%)
Worrying about my child ingesting it	5 (42%)	25 (57%)
Not wanting my child to have too much fluoride	8 (67%)	18 (41%)
Domain 3: Thinking Fluoride Is Harmful	11 (92%)	39 (89%)
Believing it is dangerous for my child’s health	3 (25%)	36 (82%)
Believing it will damage the body	8 (66%)	20 (45%)
Fearing it will affect my child’s developing mind	7 (58%)	11 (25%)
Worrying it will upset my child	6 (50%)	3 (7%)
Domain 4: Thinking There Is Too Much Uncertainty With Fluoride	9 (75%)	31 (70%)
Hearing negative things about it	7 (58%)	11 (25%)
Feeling like I don’t know enough	0 (0%)	16 (36%)
Worrying that there are unknown long term effects	2 (17%)	16 (36%)
Erring on the side of the caution	4 (33%)	10 (23%)
Domain 5: Feeling Pressured To Get Fluoride	7 (58%)	27 (61%)
Getting it pushed on me	3 (25%)	8 (18%)
Not telling me the whole truth about it	7 (58%)	13 (30%)
Feeling like there is an agenda to push fluoride	2 (17%)	18 (41%)
Domain 6: Feeling Fluoride Should Be A Choice	6 (50%)	20 (45%)
Considering my child’s opinion about it	1 (8%)	8 (18%)
Having the right to decide what is best for my child	6 (50%)	20 (45%)

#### Domain 1: Thinking Topical Fluoride Is Unnecessary

All caregivers of CSHCN (100%) believed topical fluoride was unnecessary to keep their child’s teeth healthy compared to 89% of caregivers of healthy children. A 63-year-old caregiver of a 13-year-old child with SHCN said

*He reacts to things differently than regular children…I don’t introduce anything into his world that isn’t absolutely necessary.*

Among caregivers of CSHCN, 75% believed topical fluoride is ineffective in preventing cavities compared to 59% of caregivers of healthy children. About 25% of caregivers of CSHCN compared to 50% of caregivers of healthy children believed diet is more important than topical fluoride.

#### Domain 2: Wanting to Keep Chemicals Like Fluoride Out of My Child’s Body

All caregivers of CSHCN (100%) wanted to keep chemicals like fluoride out of their child’s body compared to 82% of caregivers of healthy children. About 67% of caregivers of CSHCN expressed not wanting their child to have too much fluoride, especially if their child already received fluoride from other sources like water or toothpaste, compared to 41% of caregivers of healthy children. A 61-year-old caregiver of a 15-year-old child with SHCN said

*I would rather err on the side of caution than introduce potentially harmful chemicals into his body that may or may not disrupt his system more than it already is disrupted.*

#### Domain 3: Thinking Fluoride Is Harmful

Similar proportions of caregivers by SHCN status expressed concerns about the negative health consequences of topical fluoride (92% for CSHCN and 89% for healthy). While only 25% of caregivers of CSHCN believed fluoride is dangerous to their child’s health (compared to 82% of caregivers of healthy children), relatively larger proportions of caregivers of CSHCN believed fluoride would damage their child’s body, affect their child’s developing mind, or upset their child. A 34-year-old caregiver of a 6-year-old son with SHCN and a 12-year-old daughter with SHCN shared

*My daughter [is] very sensitive and seems to have a hormone imbalance, so I just didn’t want to continue with [topical fluoride]…[Also, my son] is already significantly delayed in his development. I can’t risk a chance that it could be hindered any further…since I was told [topical fluoride] was a neurotoxin.*

Notably, 50% of caregivers of CSHCN described their child having past negative experiences with topical fluoride compared to 7% of caregivers of healthy children. Caregivers of CSHCN expressed concerns that topical fluoride could cause their child discomfort, especially if they were sensitive to stimuli like the taste, smell, or texture of topical fluoride. A 42-year-old caregiver of an 8-year-old child with SHCN stated

*I’ve never refused treatment just because I feel differently about fluoride. It’s always how my kid’s doing that day, how they’re behaving…and if I feel like he [my son] would tolerate it.*

#### Domain 4: Thinking There Is Too Much Uncertainty with Fluoride

Similar proportions of caregivers of CSHCN and caregivers of healthy children felt there is too much uncertainty about fluoride (75% and 70%, respectively). No caregivers of CSHCN (0%) mentioned feeling that they did not know enough about fluoride (compared to 36% of caregivers of healthy children), whereas 58% had heard negative things about it (compared to 25% for healthy).

#### Domain 5: Feeling Pressured to Get Fluoride

Similar proportions of caregivers by SHCN status felt pressured to get fluoride (58% for CSHCN and 61% for healthy). Two times as many caregivers of CSHCN felt that they were not being told the whole truth about fluoride (58% for CSHCN and 30% for healthy) whereas one-half as many felt like there was an agenda on the part of others to push fluoride (17% for CSHCN and 41% for healthy).

#### Domain 6: Feeling Fluoride Should Be a Choice

About 50% of caregivers of CSHCN believed they should have autonomy over health care decisions for their child, which was comparable to the 45% of caregivers of healthy children.

## Discussion

The study goal was to understand topical fluoride-related beliefs and refusal behaviors for caregivers of CSHCN compared to caregivers of healthy children. There were three main findings. First, fluoride-related beliefs were similar for the two caregiver groups. Second, SHCN status was not significantly associated with fluoride refusal. Third, the reasons for refusal were similar but all caregivers of CSHCN thought fluoride was unnecessary and wanted to keep chemicals out of their child’s body. Collectively, our findings suggest that while caregivers of CSHCN have similar beliefs to caregivers of healthy children and are not more likely to refuse topical fluoride, there may be clinically meaningful differences in the reasons for refusing topical fluoride for caregivers of CSHCN.

Fluoride-related beliefs were similar for both groups of caregivers. A 2010 study found that caregivers of children with autism reported concerns about the safety of fluoride and its side effects (Rada, 2010). Our findings suggest that caregivers of CSHCN are not more likely to believe that topical fluoride lowers IQ or causes autism or other health problems. One potential explanation for this inconsistency is that the Rada publication focused only on caregivers of children with autism whereas we included both caregivers of CSHCN and caregivers of healthy children. Current literature suggests that vaccine related beliefs are closely related to vaccine related beliefs (Carpiano & Chi, 2018). An HPV vaccination study found that caregivers of immunosuppressed children have concerns about the side effects of vaccines, but like the Rada study, these beliefs were not compared to the beliefs of caregivers of healthy children (Seale et al, 2012). Caregivers of CSHCN may have concerns about preventive care like topical fluoride and vaccines, but more research is needed to determine whether these concerns are different.

We also found no difference in topical fluoride refusal prevalence between caregiver groups. This is consistent with a study from Singapore on silver diamine fluoride, which also found no difference in the prevalence of acceptance by autism status (Hu et al., 2020). Another study found that dentists believe caregivers of CSHCN are more likely to refuse topical fluoride, but our current findings do not support this belief (Chi & Basson, 2018). While there may not be a statistically significant difference in topical fluoride refusal by SHCN status, it is important to note that the prevalence of topical fluoride hesitancy and refusal among caregivers of CSHCN in our study was high (34.5%). Given that caregivers of CSHCN are more likely to report their child to have poor oral health, not getting topical fluoride may have disproportionately negative consequences for children at higher risk for tooth decay. Future research should assess whether topical fluoride refusal leads to poorer health outcomes for CSHCN compared to healthy children.

We also found that reasons for refusal were similar across caregiver groups, with two notable differences. All interviewed caregivers of CSHCN thought that fluoride was unnecessary and wanted to keep chemicals out of their child’s body. Vaccine hesitancy studies on caregivers of CSHCN have also cited concerns about toxins and vaccines being unnecessary as reasons why caregivers refuse vaccines for their child (Hofstetter et al., 2018; Mensah-Bonsu et al., 2021). Although the literature implies that the six-domain conceptual model of topical fluoride hesitancy applies to all caregivers, our findings suggest that the reasons underlying refusal are nuanced (Chi et al., 2023). Future work should focus on validating our findings to determine if the conceptual model is applicable to subgroups of caregivers.

While additional research is needed to understand how caregivers of CSHCN make decisions about topical fluoride, our study findings have immediate clinical relevance. Several chairside strategies can be used to address topical fluoride hesitancy and refusal including the following: (1) assess the child’s tooth decay risk; (2) determine caregiver’s beliefs about topical fluoride and reasons for refusal or hesitancy; (3) ask about willingness to try other forms of fluoride and alternative prevention strategies, such as dietary changes (Chi, 2017).

There were two main study limitations. First, we recruited a small number of caregivers of CSHCN. Future studies should focus on recruiting large groups of caregivers of CSHCN to assess the validity of our findings. Second, because of the heterogeneity within the CSHCN population, our screening question for CSHCN in the quantitative survey may have led to misclassification. We did not collect specific diagnostic information about the child. However, the prevalence of CSHCN in our study is consistent with previously reported national prevalence of CSHCN, which is an indication that our SHCN measure is accurate. Future studies should focus on identifying specific subgroups at high risk for dental caries, such as children with intellectual or developmental disabilities.

## Conclusions for Practice

While caregivers of CSHCN had similar beliefs and were equally as likely to refuse topical fluoride as caregivers of healthy children, they may have different reasons for refusing topical fluoride. These reasons are important in guiding the development of tailored interventions for caregivers of CSHCN, which can ultimately help to address oral health disparities experienced by children who have the most to benefit from the preventive effects of topical fluoride.

### Electronic supplementary material

Below is the link to the electronic supplementary material.

Appendix A: Survey demographics included caregiver age, gender, race, ethnicity, education, child SHCN status, and household income.

## Data Availability

The data used in this manuscript are not publicly available.

## Abbreviations

CSHCN: Children with special health care needs
SHCN: Special health care needs
ASD: Autism spectrum disorder
OR: Odds ratios
AOR: Adjusted odds ratios
