# Supplementary figures and images for: A Mixed-Methods Study on Topical Fluoride Beliefs and Refusal Behaviors for Caregivers of Children with Special Health Care Needs
Source: Matern Child Health J. 2023 Nov 15;28(1):104–15. doi: 10.1007/s10995-023-03806-1 (PMC10876822; doi:10.1007/s10995-023-03806-1)

**Appendix A**

**
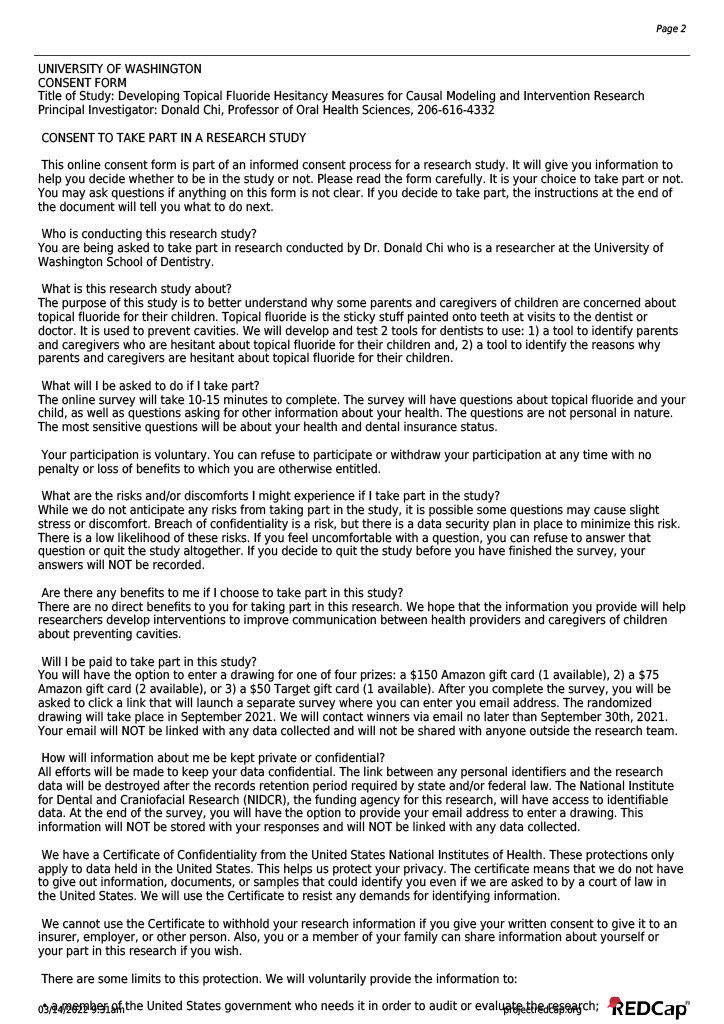
**85 item caregiver survey

**
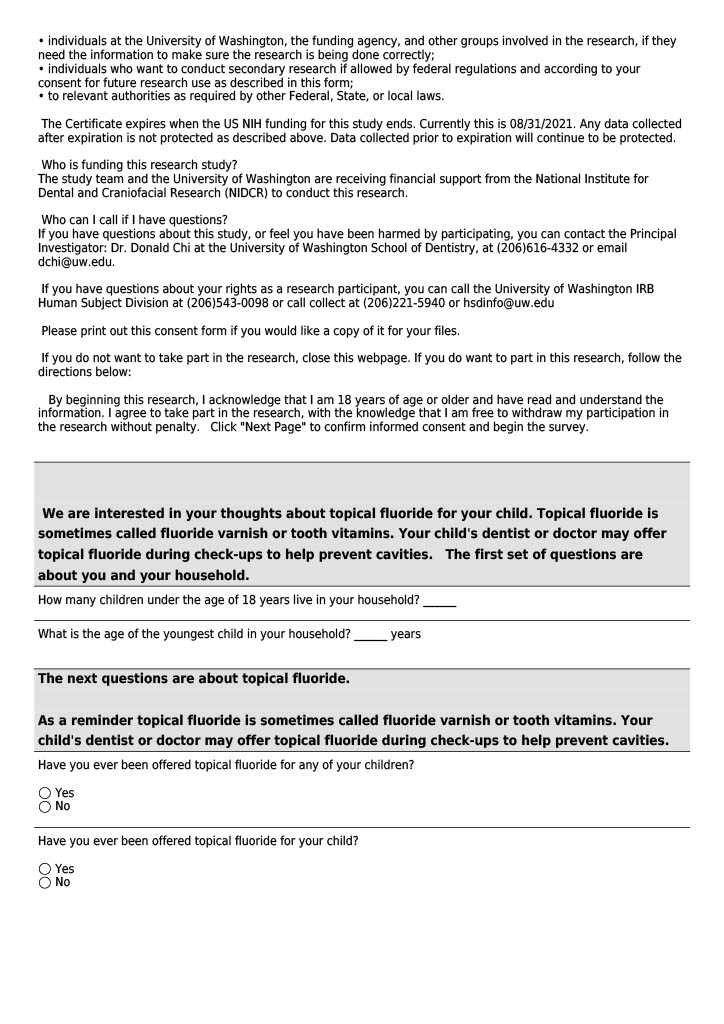

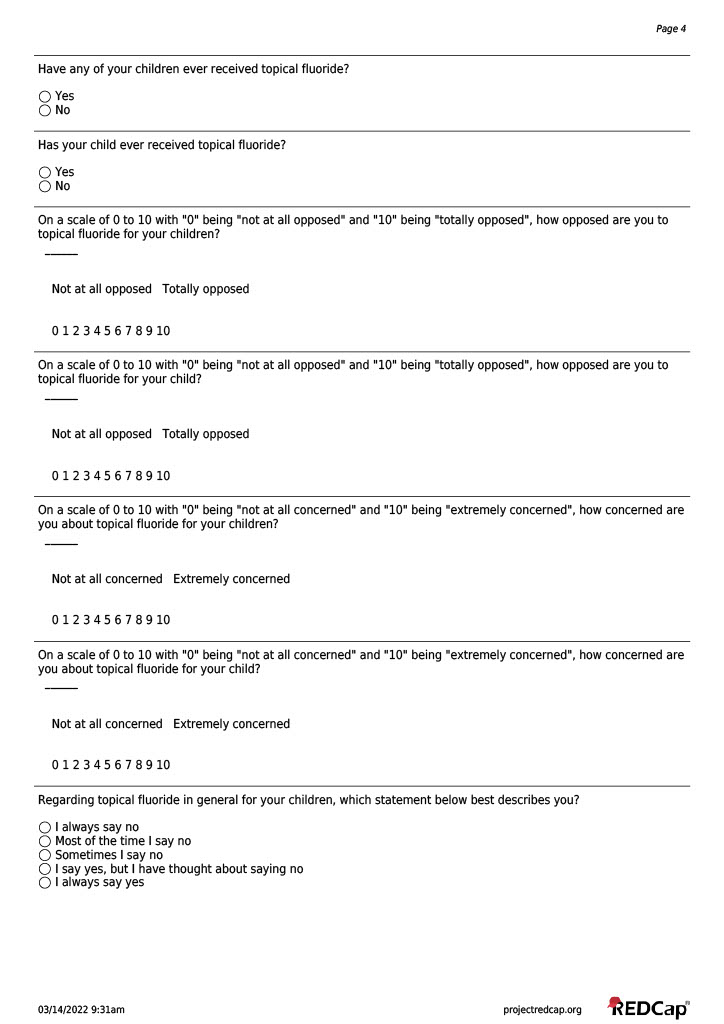

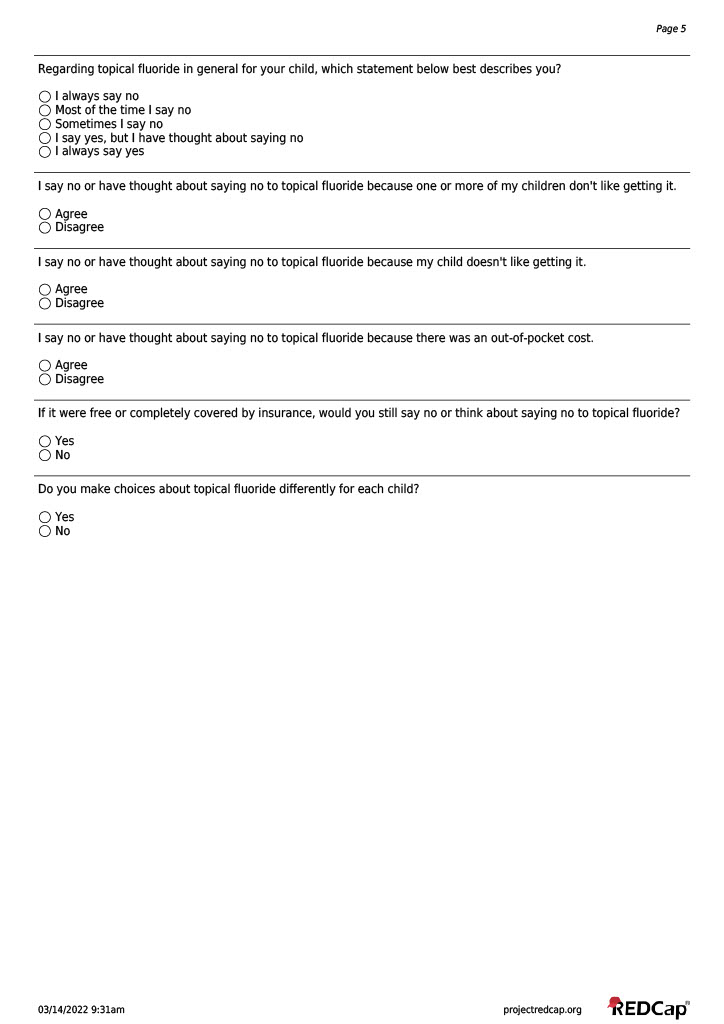

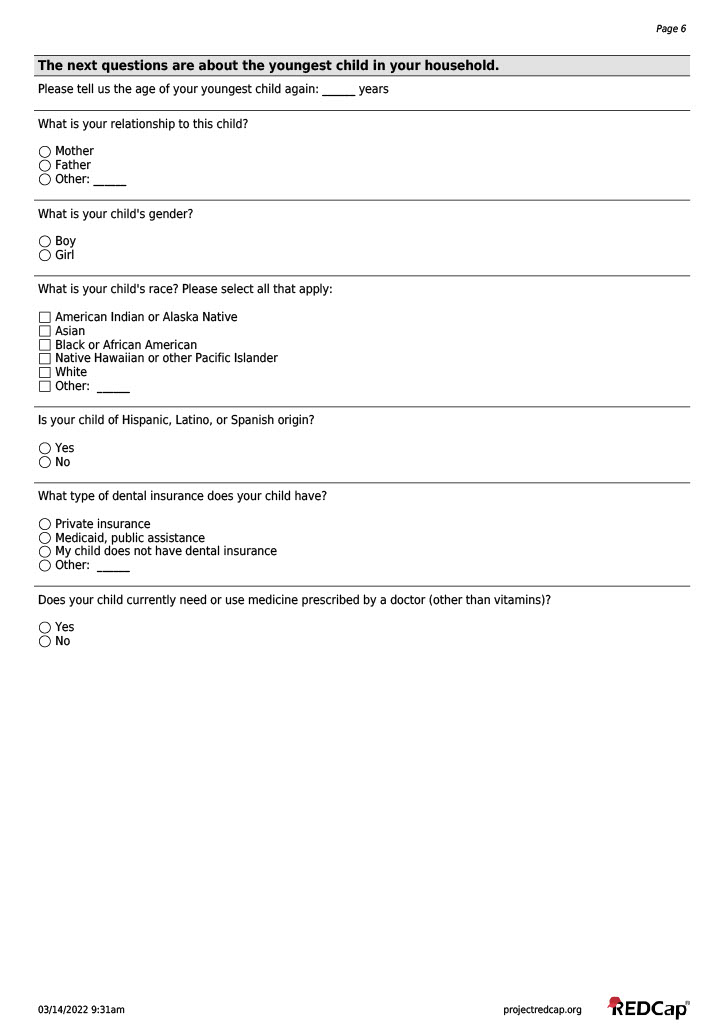

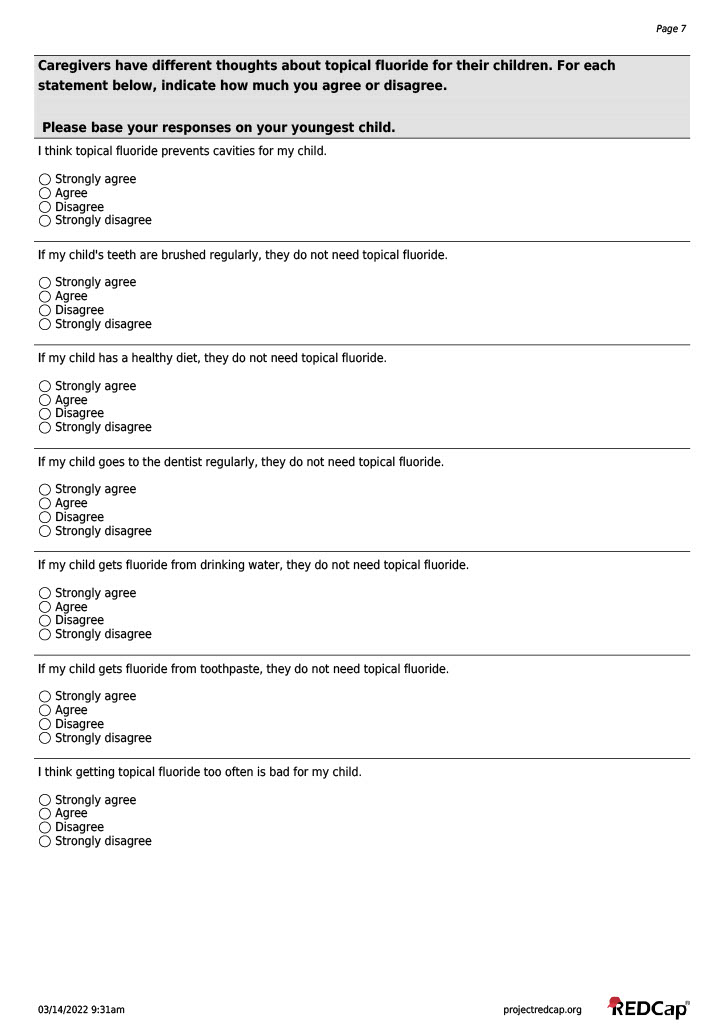

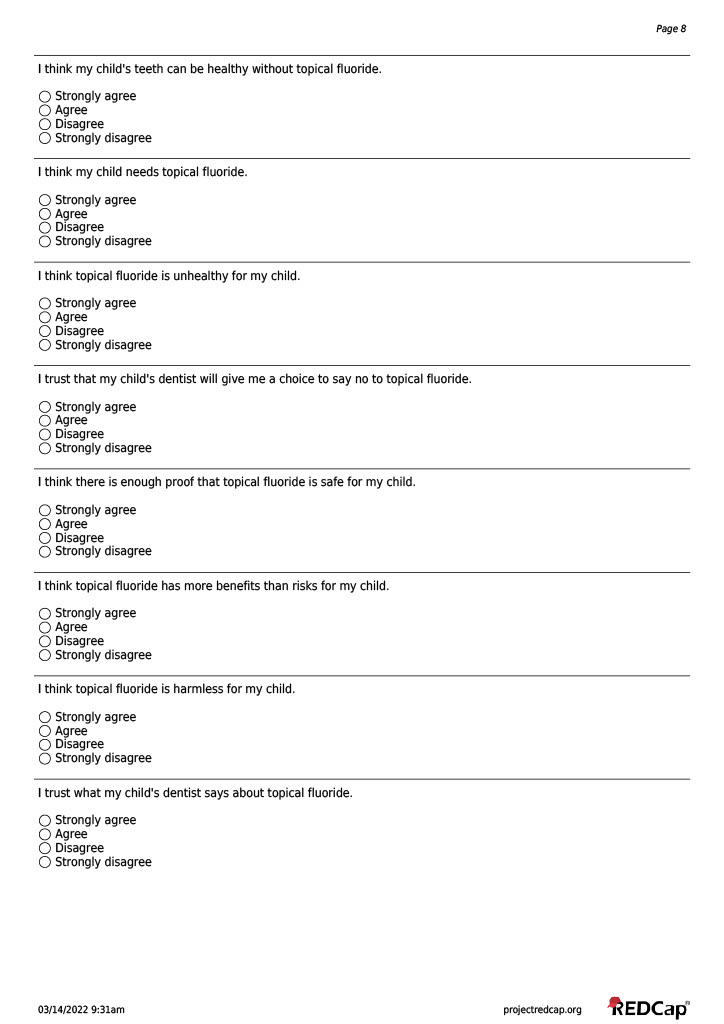

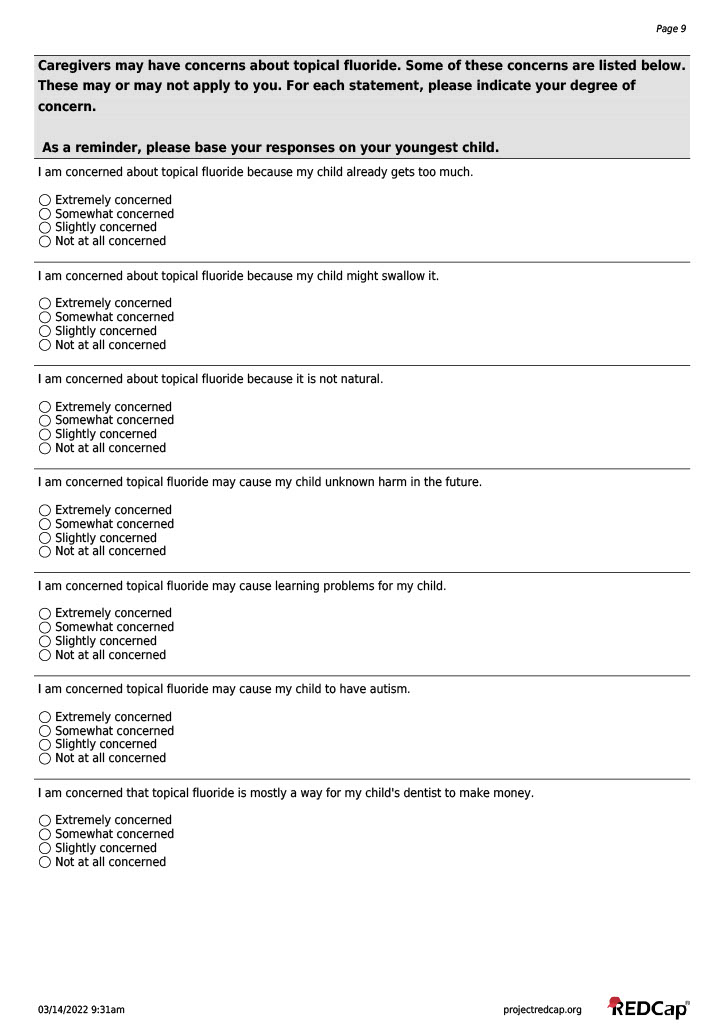

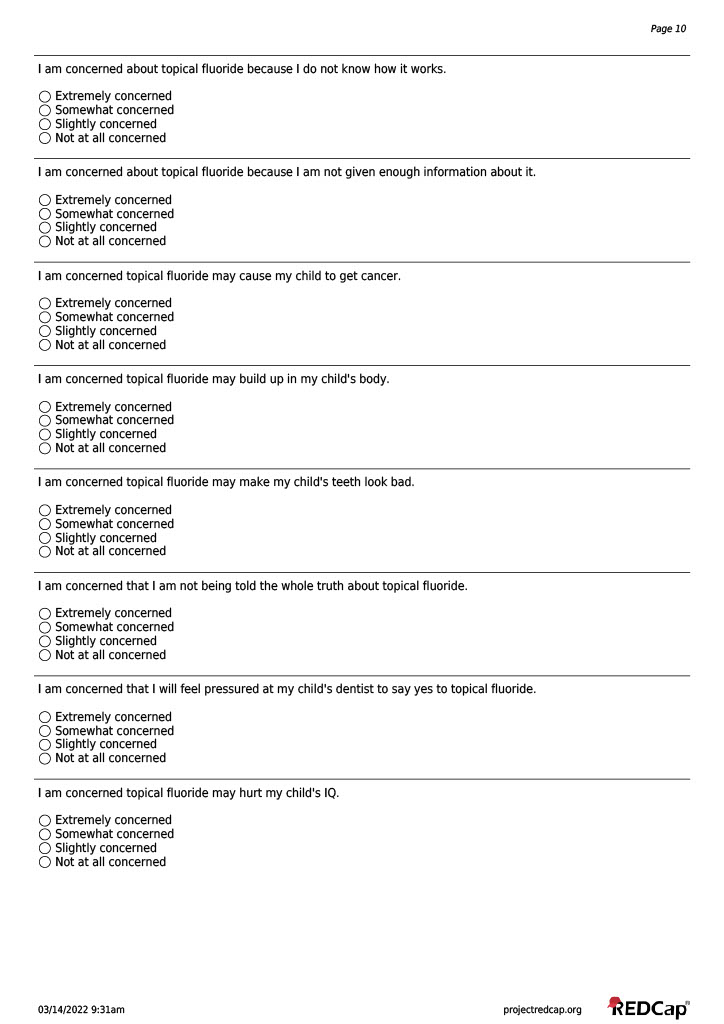

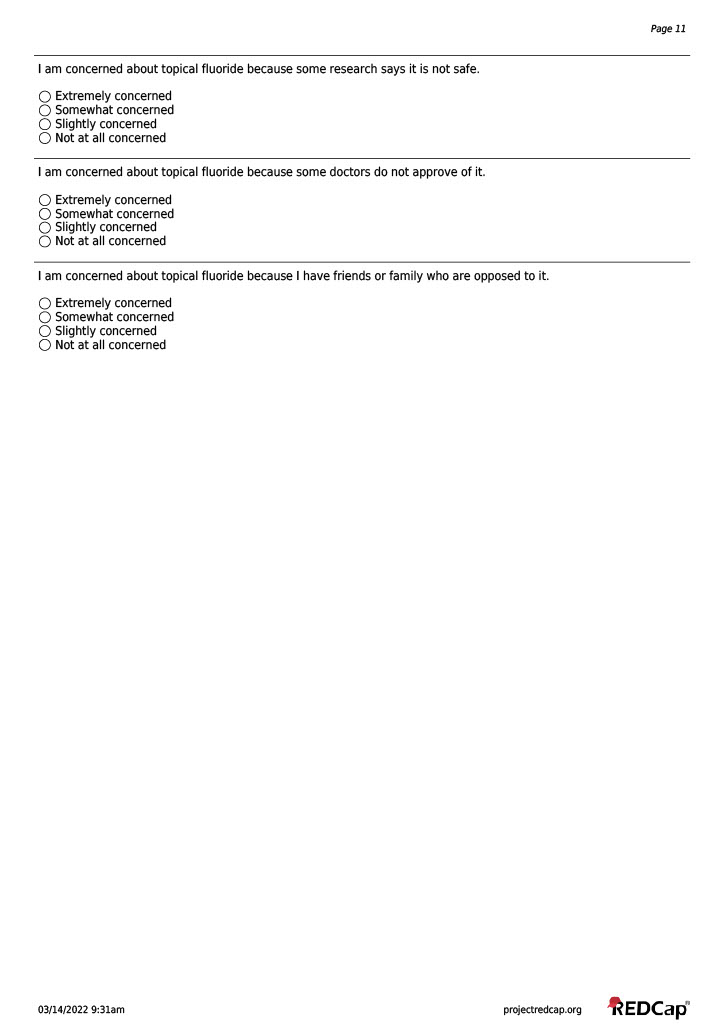

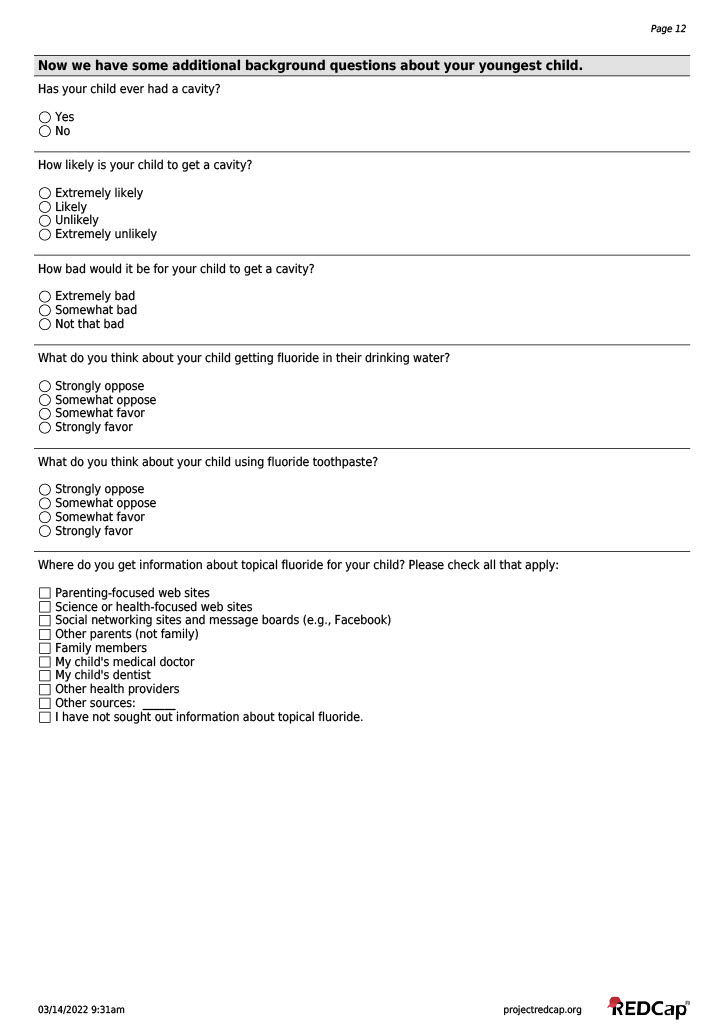

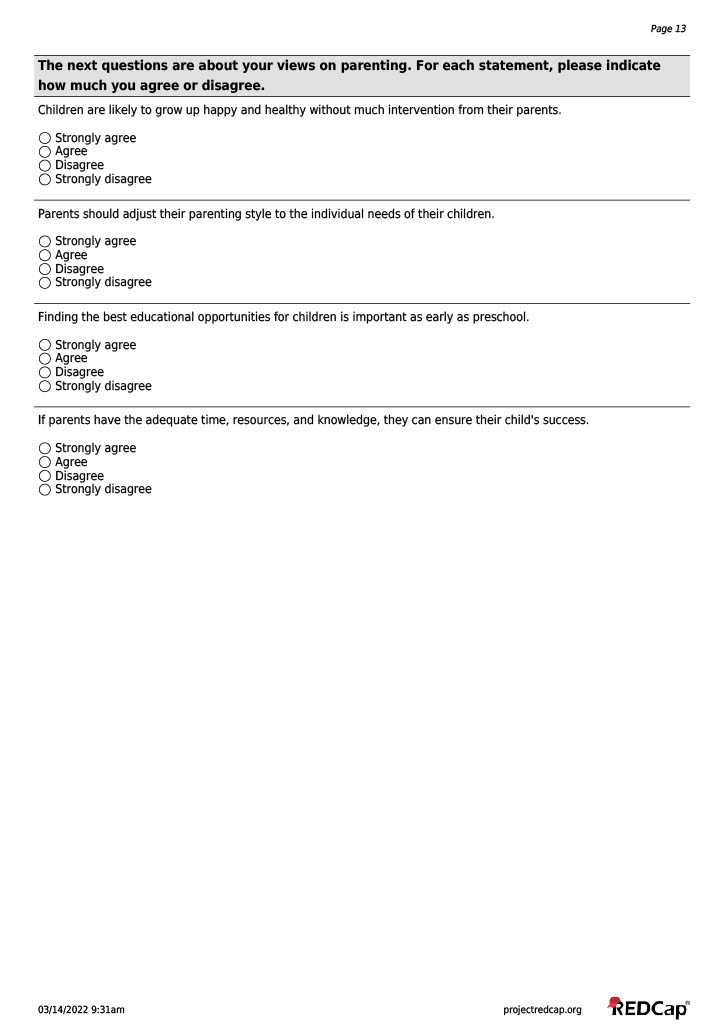

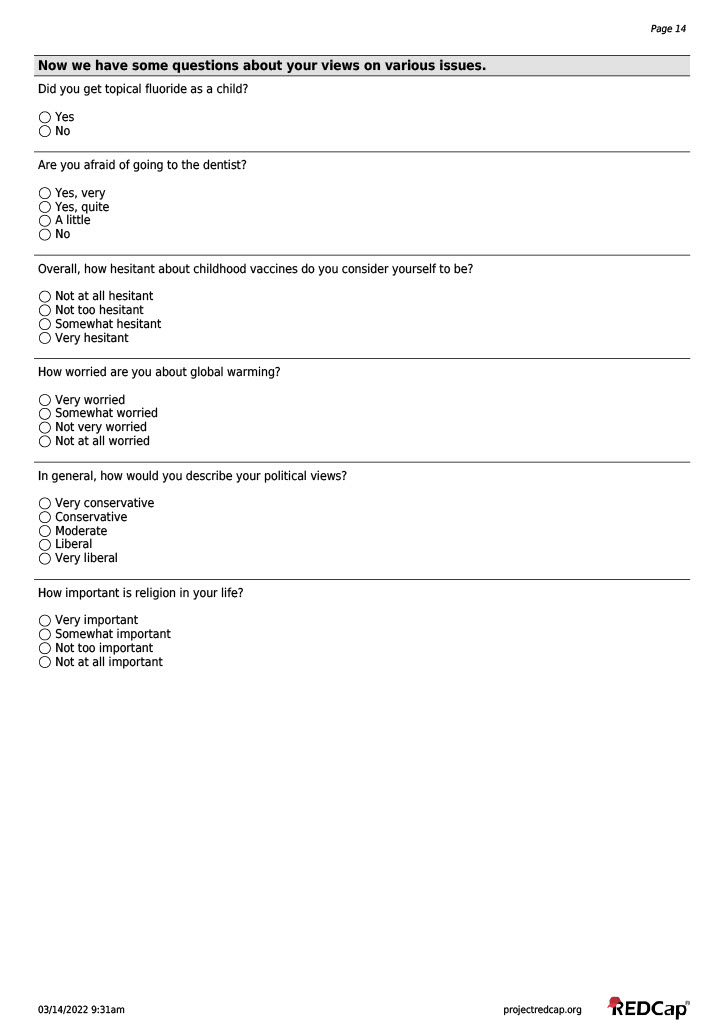

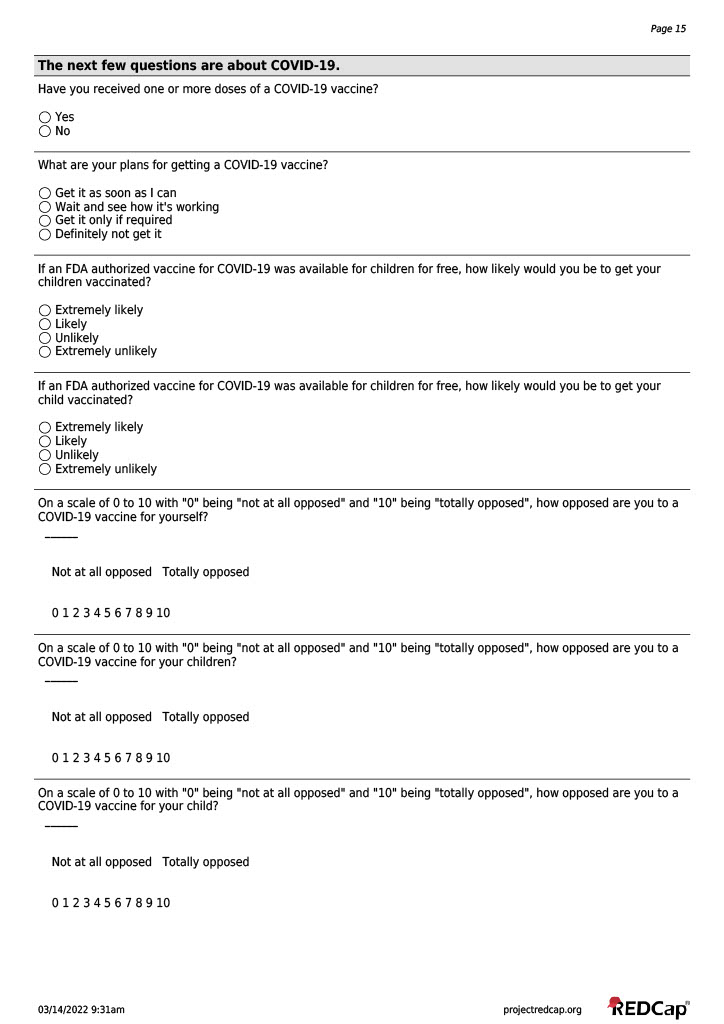

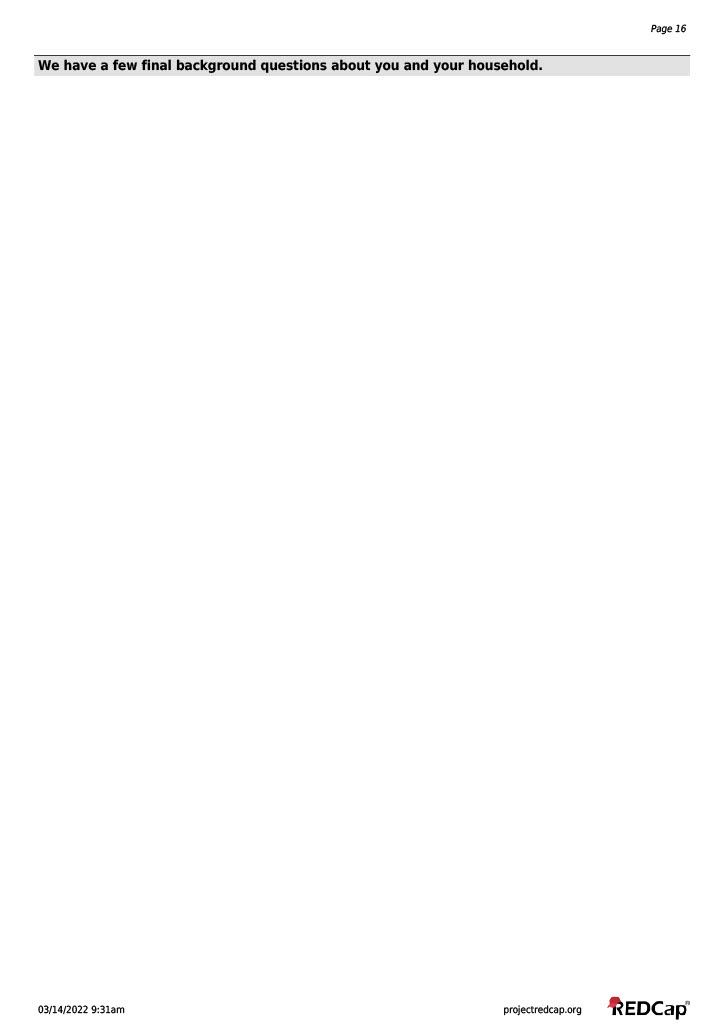

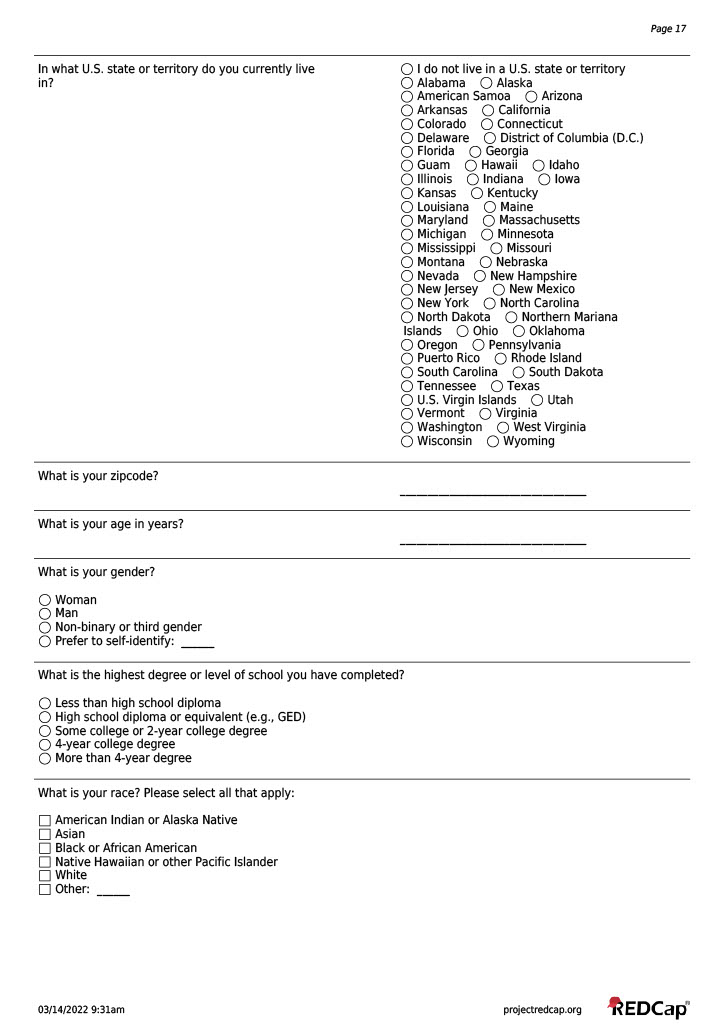

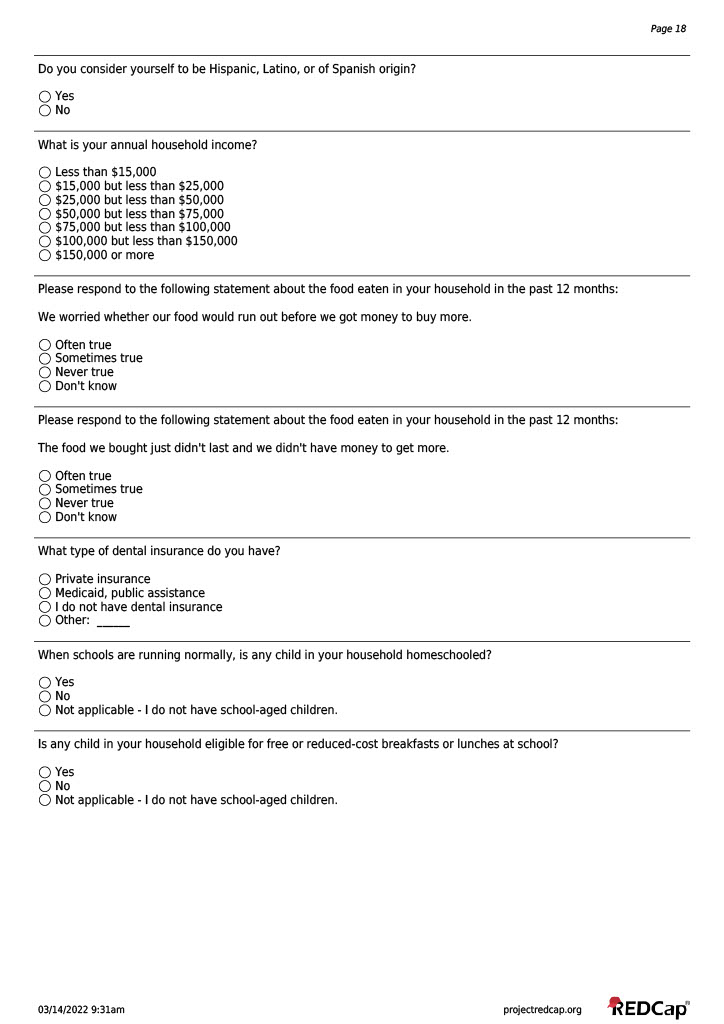

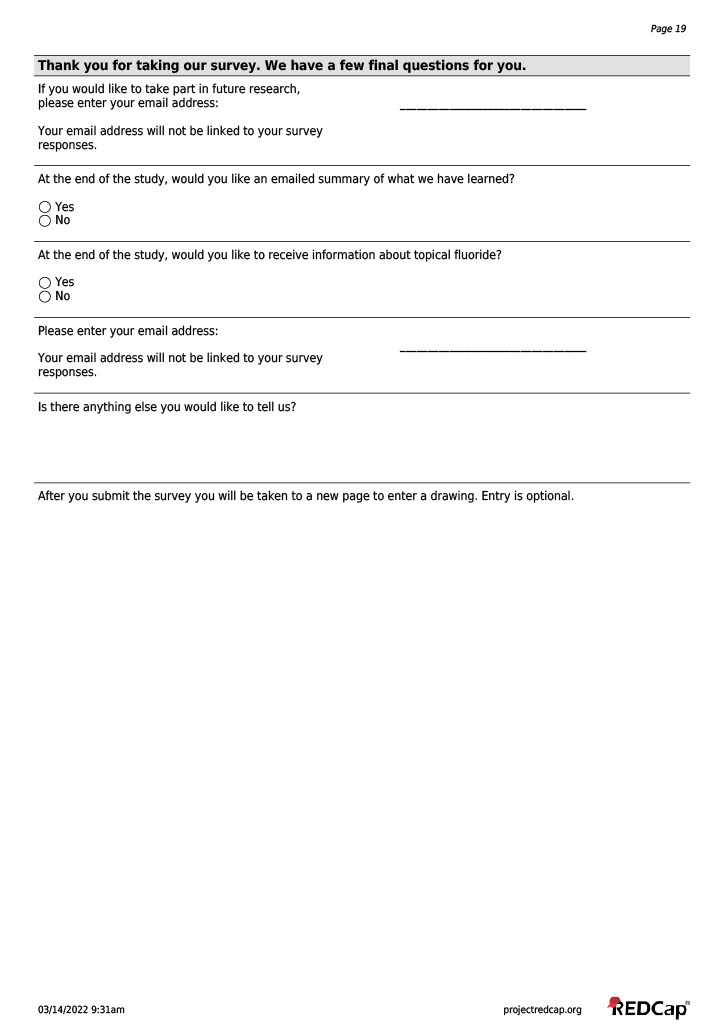
**

Supplement: Supplementary file 1 — Survey demographics included caregiver age, gender, race, ethnicity, education, child SHCN status, and household income. [file 10995_2023_3806_MOESM1_ESM.docx]
